# *Tert*-butyl 3-oxo-2,3,4,5,6,7-hexa­hydro-1*H*-pyrazolo[4,3-*c*]pyridine-5-carboxyl­ate

**DOI:** 10.1107/S1600536809053021

**Published:** 2009-12-16

**Authors:** Tara Shahani, Hoong-Kun Fun, R. Venkat Ragavan, V. Vijayakumar, S. Sarveswari

**Affiliations:** a X-ray Crystallography Unit, School of Physics, Universiti Sains Malaysia, 11800 USM, Penang, Malaysia; b Organic Chemistry Division, School of Advanced Sciences, VIT University, Vellore 632 014, India

## Abstract

In the title compound, C_11_H_17_N_3_O_3_, the pyrazole ring is approximately planar, with a maximum deviation of 0.005 (2) Å, and forms a dihedral angle of 5.69 (13)° with the plane through the six atoms of the piperidine ring. In the crystal, pairs of inter­molecular N—H⋯O hydrogen bonds form dimers with neighbouring mol­ecules, generating *R*_2_^2^(8) ring motifs. These dimers are further linked into two-dimensional arrays parallel to the *bc* plane by inter­molecular N—H⋯O and C—H⋯O hydrogen bonds.

## Related literature

For the biological activity of pyrazolone derivatives, see: Al-Haiza *et al.* (2001 ▶); Brogden, (1986 ▶); Coersmeier *et al.* (1986 ▶); Gursoy *et al.* (2000 ▶). For myocardial ischemia, see: Wu *et al.* (2002 ▶). For brain ischemia, see: Watanabe *et al.* (1984 ▶); Kawai *et al.* (1997 ▶). For new compounds with the pyrazolone unit, see: Al-Haiza *et al.* (2001 ▶). For a related structure, see: Shahani *et al.* (2009 ▶). For ring conformations, see: Cremer & Pople (1975 ▶). For hydrogen-bond motifs, see: Bernstein *et al.* (1995 ▶). For bond-length data, see: Allen *et al.* (1987 ▶). For the stability of the temperature controller used for the data collection, see: Cosier & Glazer (1986 ▶).
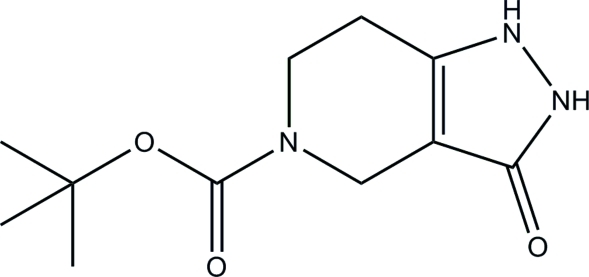


## Experimental

### 

#### Crystal data

C_11_H_17_N_3_O_3_
*M* *_r_* = 239.28
Monoclinic, 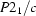

*a* = 18.6250 (12) Å
*b* = 6.0893 (5) Å
*c* = 10.7414 (7) Å
β = 104.100 (4)°
*V* = 1181.51 (15) Å^3^
*Z* = 4
Mo *K*α radiation
μ = 0.10 mm^−1^
*T* = 100 K
0.97 × 0.35 × 0.14 mm

#### Data collection

Bruker SMART APEXII CCD area-detector diffractometer
Absorption correction: multi-scan (*SADABS*; Bruker, 2009 ▶) *T*_min_ = 0.910, *T*_max_ = 0.986
13101 measured reflections
2690 independent reflections
2136 reflections with *I* > 2σ(*I*)
*R*_int_ = 0.035

#### Refinement

*R*[*F* ^2^ > 2σ(*F* ^2^)] = 0.054
*wR*(*F* ^2^) = 0.172
*S* = 1.24
2690 reflections
222 parameters
All H-atom parameters refined
Δρ_max_ = 0.39 e Å^−3^
Δρ_min_ = −0.31 e Å^−3^

### 

Data collection: *APEX2* (Bruker, 2009 ▶); cell refinement: *SAINT* (Bruker, 2009 ▶); data reduction: *SAINT*; program(s) used to solve structure: *SHELXTL* (Sheldrick, 2008 ▶); program(s) used to refine structure: *SHELXTL*; molecular graphics: *SHELXTL*; software used to prepare material for publication: *SHELXTL* and *PLATON* (Spek, 2009 ▶).

## Supplementary Material

Crystal structure: contains datablocks global, I. DOI: 10.1107/S1600536809053021/ng2705sup1.cif

Structure factors: contains datablocks I. DOI: 10.1107/S1600536809053021/ng2705Isup2.hkl

Additional supplementary materials:  crystallographic information; 3D view; checkCIF report

## Figures and Tables

**Table 1:** Hydrogen-bond geometry (Å, °)

*D*—H⋯*A*	*D*—H	H⋯*A*	*D*⋯*A*	*D*—H⋯*A*
N1—H1*N*1⋯O2^i^	0.99 (3)	1.77 (3)	2.748 (3)	171 (3)
N2—H1*N*2⋯O2^ii^	0.94 (3)	1.75 (4)	2.665 (3)	167 (3)
C1—H1*B*⋯O2^iii^	0.98 (3)	2.57 (3)	3.492 (3)	157 (3)
C11—H11*C*⋯O3^iv^	0.97 (3)	2.60 (3)	3.504 (3)	156 (3)

Symmetry codes: (i) 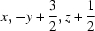; (ii) 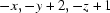; (iii) 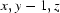; (iv) 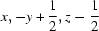.

**Fig. 1.**
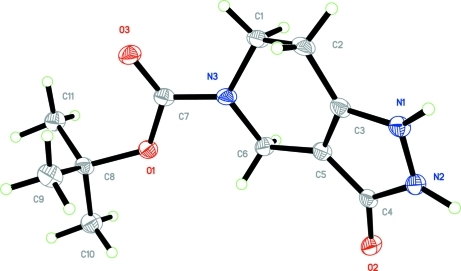
The molecular structure of the title compound, showing 50% probability displacement ellipsoids and the atom numbering scheme.

**Fig. 2.**
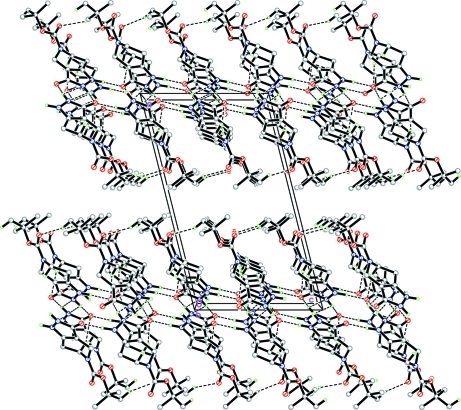
The crystal packing of the title compound, viewed along b axis, showing two-dimensional arrays parallel to the bc plane.

## supplementary crystallographic information

### Comment

Pyrazolone derivatives have a broad spectrum of biological activities being used as analgesic, antipyretic and anti-inflammatory therapeutical drugs (Brogden, 1986; Gursoy *et al.*, 2000). A class of new compounds with pyrazolone moiety was synthesized and reported for their antibacterial, antifungal activities by Al-Haiza *et al.* (2001). A new pyrazolone derivative, edaravone (3-methyl-1-phenyl-2-pyrazoline-5-one) is being used as a drug in clinical practice for brain ischemia (Watanabe *et al.*, 1984; Kawai *et al.*, 1997) and the same has been found to be effective against myocardial ischemia (Wu *et al.*, 2002).

In the crystal structure (Fig. 1), the pyrazole ring (C3/N1/N2/C4/C5) is approximately planar, with a maximum deviation of 0.005 (2) Å at atom N2. The piperidine ring (C1/C2/C3/C5/C6/N3) adopts a half-boat conformation (Cremer & Pople, 1975) with puckering of Q = 0.465 (2) Å, Θ = 52.9 (2)° & φ = 39.8 (4)°. The maximum deviation in this piperidine ring is 0.286 Å at atom N3. The dihedral angle formed between the mean planes of pyrazole and piperidine rings is 5.69 (13)°. The bond lengths (Allen *et al.*, 1987) and angles are within normal ranges and comparable to a closely related structure (Shahani *et al.*, 2009).

In the crystal packing (Fig. 2), pairs of intermolecular N2—H1N2···O2 hydrogen bonds form dimers with neighbouring molecules, generating *R*^2^_2_(8) ring motifs (Bernstein *et al.*, 1995). These dimers are further linked into two-dimensional arrays parallel to the *bc* plane by intermolecular N1—H1N1···O2, C1—H1B···O2 and C11—H11C···O3 hydrogen bonds.

### Experimental

LiHMDS (1.0 *M* solution in toluene, 11 mmol) was added quickly to a solution of *tert*-butyl 4-oxopiperidine-1-carboxylate (10 mmol 15 ml of toluene) using syringe at 273 K with stirring for 10 minutes. Ethyl chloro formate (11 mmol) was then added quickly. The reaction mixture was slowly (10 minutes) brought to room temperature and stirred for 10 minutes. Acetic acid (2 ml), ethanol (15 ml), and hydrazine hydrate (30 mmol) were added and refluxed for 15 minutes. The reaction mixture was concentrated to dryness under reduced pressure and re-dissolved in ethyl acetate. The organic layer was washed with saturated brine solution, dried over Na_2_SO_4_, evaporated under reduced pressure and purified by crystallizing using ethanol (white solid). The recrystallization was done using 1:1 mixture of ethanol and acetone. Yield: 78%. M.p. 498.5–500.5 K. MS calculated for C_11_H_17_N_3_O_3_: 239.126. Found: 239.80 (*M*+).

### Refinement

All hydrogen atoms were located in a difference map and were refined freely [range of N—H = 0.94 and 0.99 Å; and C—H = 0.95–1.02 Å].

### Crystal data

**Table:**

C_11_H_17_N_3_O_3_	*F*(000) = 512
*M**_r_* = 239.28	*D*_x_ = 1.345 Mg m^−^^3^
Monoclinic, *P*2_1_/*c*	Mo *K*α radiation, λ = 0.71073 Å
Hall symbol: -P 2ybc	Cell parameters from 7287 reflections
*a* = 18.6250 (12) Å	θ = 2.3–33.5°
*b* = 6.0893 (5) Å	µ = 0.10 mm^−^^1^
*c* = 10.7414 (7) Å	*T* = 100 K
β = 104.100 (4)°	Plate, colourless
*V* = 1181.51 (15) Å^3^	0.97 × 0.35 × 0.14 mm
*Z* = 4	

### Data collection

**Table:**

Bruker SMART APEXII CCD area-detector diffractometer	2690 independent reflections
Radiation source: fine-focus sealed tube	2136 reflections with *I* > 2σ(*I*)
graphite	*R*_int_ = 0.035
φ and ω scans	θ_max_ = 27.5°, θ_min_ = 1.1°
Absorption correction: multi-scan (*SADABS*; Bruker, 2009)	*h* = −24→24
*T*_min_ = 0.910, *T*_max_ = 0.986	*k* = −7→7
13101 measured reflections	*l* = −13→13

### Refinement

**Table:**

Refinement on *F*^2^	Primary atom site location: structure-invariant direct methods
Least-squares matrix: full	Secondary atom site location: difference Fourier map
*R*[*F*^2^ > 2σ(*F*^2^)] = 0.054	Hydrogen site location: inferred from neighbouring sites
*wR*(*F*^2^) = 0.172	All H-atom parameters refined
*S* = 1.24	*w* = 1/[σ^2^(*F*_o_^2^) + (0.0688*P*)^2^ + 1.2823*P*] where *P* = (*F*_o_^2^ + 2*F*_c_^2^)/3
2690 reflections	(Δ/σ)_max_ < 0.001
222 parameters	Δρ_max_ = 0.39 e Å^−^^3^
0 restraints	Δρ_min_ = −0.31 e Å^−^^3^

### Special details

**Table:**

Experimental. The crystal was placed in the cold stream of an Oxford Cyrosystems Cobra open-flow nitrogen cryostat (Cosier & Glazer, 1986) operating at 100.0 (1) K.
Geometry. All e.s.d.'s (except the e.s.d. in the dihedral angle between two l.s. planes) are estimated using the full covariance matrix. The cell e.s.d.'s are taken into account individually in the estimation of e.s.d.'s in distances, angles and torsion angles; correlations between e.s.d.'s in cell parameters are only used when they are defined by crystal symmetry. An approximate (isotropic) treatment of cell e.s.d.'s is used for estimating e.s.d.'s involving l.s. planes.
Refinement. Refinement of *F*^2^ against ALL reflections. The weighted *R*-factor *wR* and goodness of fit *S* are based on *F*^2^, conventional *R*-factors *R* are based on *F*, with *F* set to zero for negative *F*^2^. The threshold expression of *F*^2^ > σ(*F*^2^) is used only for calculating *R*-factors(gt) *etc*. and is not relevant to the choice of reflections for refinement. *R*-factors based on *F*^2^ are statistically about twice as large as those based on *F*, and *R*- factors based on ALL data will be even larger.

### Fractional atomic coordinates and isotropic or equivalent isotropic displacement parameters (Å^2^)

**Table:**

	*x*	*y*	*z*	*U*_iso_*/*U*_eq_	
O1	0.30793 (9)	0.5995 (3)	0.33483 (16)	0.0205 (4)	
O2	0.04609 (9)	0.9437 (3)	0.36561 (15)	0.0205 (4)	
O3	0.34752 (9)	0.2971 (3)	0.45801 (16)	0.0231 (4)	
N1	0.07659 (11)	0.5955 (4)	0.62882 (18)	0.0191 (4)	
N2	0.04170 (11)	0.7682 (4)	0.55702 (19)	0.0186 (4)	
N3	0.23023 (11)	0.4313 (3)	0.43120 (19)	0.0191 (4)	
C1	0.20586 (14)	0.2460 (4)	0.4972 (2)	0.0215 (5)	
C2	0.17706 (14)	0.3233 (4)	0.6127 (2)	0.0225 (5)	
C3	0.12708 (12)	0.5145 (4)	0.5701 (2)	0.0185 (5)	
C4	0.06920 (12)	0.7945 (4)	0.4514 (2)	0.0167 (5)	
C5	0.12428 (12)	0.6319 (4)	0.4598 (2)	0.0172 (5)	
C6	0.17364 (13)	0.5912 (4)	0.3715 (2)	0.0190 (5)	
C7	0.30029 (12)	0.4308 (4)	0.4123 (2)	0.0180 (5)	
C8	0.38126 (12)	0.6603 (4)	0.3140 (2)	0.0186 (5)	
C9	0.43236 (15)	0.7313 (5)	0.4403 (3)	0.0258 (6)	
C10	0.36107 (14)	0.8534 (5)	0.2231 (3)	0.0249 (6)	
C11	0.41358 (14)	0.4723 (5)	0.2519 (2)	0.0215 (5)	
H1A	0.2501 (16)	0.144 (5)	0.527 (3)	0.027 (7)*	
H1B	0.1645 (16)	0.173 (5)	0.437 (3)	0.025 (7)*	
H2A	0.1514 (16)	0.203 (6)	0.639 (3)	0.032 (8)*	
H2B	0.2187 (16)	0.364 (5)	0.682 (3)	0.026 (7)*	
H6A	0.1980 (15)	0.727 (5)	0.353 (3)	0.023 (7)*	
H6B	0.1445 (14)	0.539 (5)	0.286 (3)	0.021 (7)*	
H9A	0.4124 (16)	0.854 (5)	0.476 (3)	0.026 (8)*	
H9B	0.4808 (16)	0.779 (5)	0.428 (3)	0.026 (7)*	
H9C	0.4426 (16)	0.611 (6)	0.504 (3)	0.035 (9)*	
H10A	0.4080 (17)	0.909 (5)	0.201 (3)	0.035 (8)*	
H10B	0.3254 (17)	0.809 (5)	0.139 (3)	0.033 (8)*	
H10C	0.3394 (19)	0.970 (6)	0.264 (3)	0.049 (10)*	
H11A	0.4596 (16)	0.530 (5)	0.227 (3)	0.031 (8)*	
H11B	0.4299 (15)	0.344 (5)	0.313 (3)	0.027 (7)*	
H11C	0.3819 (18)	0.421 (6)	0.172 (3)	0.042 (9)*	
H1N1	0.0676 (18)	0.566 (6)	0.714 (3)	0.043 (9)*	
H1N2	0.0081 (18)	0.852 (6)	0.589 (3)	0.046 (10)*	

### Atomic displacement parameters (Å^2^)

**Table:**

	*U*^11^	*U*^22^	*U*^33^	*U*^12^	*U*^13^	*U*^23^
O1	0.0230 (8)	0.0192 (9)	0.0230 (8)	0.0028 (7)	0.0124 (6)	0.0066 (7)
O2	0.0288 (8)	0.0201 (9)	0.0164 (8)	0.0054 (7)	0.0128 (6)	0.0019 (7)
O3	0.0267 (8)	0.0206 (10)	0.0245 (9)	0.0053 (7)	0.0109 (7)	0.0061 (8)
N1	0.0267 (10)	0.0186 (11)	0.0152 (9)	−0.0001 (8)	0.0111 (7)	0.0008 (8)
N2	0.0246 (9)	0.0184 (11)	0.0160 (9)	0.0018 (8)	0.0110 (7)	0.0002 (8)
N3	0.0250 (10)	0.0148 (11)	0.0210 (10)	0.0028 (8)	0.0121 (8)	0.0043 (9)
C1	0.0285 (12)	0.0144 (12)	0.0253 (12)	0.0000 (10)	0.0138 (10)	0.0016 (11)
C2	0.0299 (12)	0.0177 (13)	0.0237 (12)	0.0001 (10)	0.0134 (10)	0.0069 (11)
C3	0.0235 (11)	0.0153 (12)	0.0192 (11)	−0.0036 (9)	0.0103 (9)	−0.0027 (10)
C4	0.0212 (10)	0.0163 (12)	0.0153 (10)	−0.0027 (9)	0.0099 (8)	−0.0018 (9)
C5	0.0224 (10)	0.0151 (12)	0.0164 (10)	−0.0015 (9)	0.0094 (8)	−0.0019 (9)
C6	0.0259 (11)	0.0182 (13)	0.0166 (11)	0.0041 (10)	0.0121 (9)	0.0014 (10)
C7	0.0244 (11)	0.0150 (12)	0.0167 (10)	0.0001 (10)	0.0089 (8)	−0.0012 (10)
C8	0.0222 (11)	0.0162 (12)	0.0213 (11)	−0.0014 (9)	0.0127 (9)	0.0010 (10)
C9	0.0327 (13)	0.0248 (15)	0.0224 (13)	−0.0028 (11)	0.0115 (10)	−0.0041 (12)
C10	0.0298 (12)	0.0237 (14)	0.0250 (13)	0.0024 (11)	0.0140 (10)	0.0053 (11)
C11	0.0265 (12)	0.0208 (13)	0.0198 (12)	0.0023 (10)	0.0106 (9)	−0.0032 (11)

### Geometric parameters (Å, °)

**Table:**

O1—C7	1.351 (3)	C3—C5	1.374 (3)
O1—C8	1.484 (3)	C4—C5	1.413 (3)
O2—C4	1.290 (3)	C5—C6	1.494 (3)
O3—C7	1.212 (3)	C6—H6A	0.98 (3)
N1—C3	1.347 (3)	C6—H6B	1.00 (3)
N1—N2	1.371 (3)	C8—C10	1.516 (4)
N1—H1N1	0.99 (3)	C8—C9	1.518 (4)
N2—C4	1.364 (3)	C8—C11	1.521 (3)
N2—H1N2	0.94 (4)	C9—H9A	0.96 (3)
N3—C7	1.369 (3)	C9—H9B	0.99 (3)
N3—C1	1.462 (3)	C9—H9C	0.99 (3)
N3—C6	1.463 (3)	C10—H10A	1.02 (3)
C1—C2	1.540 (3)	C10—H10B	1.02 (3)
C1—H1A	1.02 (3)	C10—H10C	0.97 (4)
C1—H1B	0.98 (3)	C11—H11A	1.02 (3)
C2—C3	1.491 (4)	C11—H11B	1.02 (3)
C2—H2A	0.95 (3)	C11—H11C	0.97 (3)
C2—H2B	0.97 (3)		
			
C7—O1—C8	121.42 (18)	N3—C6—H6A	109.1 (16)
C3—N1—N2	107.86 (19)	C5—C6—H6A	111.8 (17)
C3—N1—H1N1	132 (2)	N3—C6—H6B	111.4 (16)
N2—N1—H1N1	120 (2)	C5—C6—H6B	110.9 (15)
C4—N2—N1	109.62 (19)	H6A—C6—H6B	105 (2)
C4—N2—H1N2	132 (2)	O3—C7—O1	125.9 (2)
N1—N2—H1N2	118 (2)	O3—C7—N3	124.4 (2)
C7—N3—C1	119.4 (2)	O1—C7—N3	109.70 (19)
C7—N3—C6	123.2 (2)	O1—C8—C10	101.39 (18)
C1—N3—C6	116.77 (19)	O1—C8—C9	109.63 (18)
N3—C1—C2	111.4 (2)	C10—C8—C9	111.0 (2)
N3—C1—H1A	107.4 (17)	O1—C8—C11	110.8 (2)
C2—C1—H1A	110.2 (16)	C10—C8—C11	111.3 (2)
N3—C1—H1B	108.7 (17)	C9—C8—C11	112.2 (2)
C2—C1—H1B	107.2 (16)	C8—C9—H9A	111.1 (17)
H1A—C1—H1B	112 (2)	C8—C9—H9B	110.9 (16)
C3—C2—C1	107.8 (2)	H9A—C9—H9B	106 (2)
C3—C2—H2A	111.7 (19)	C8—C9—H9C	112.3 (19)
C1—C2—H2A	107.4 (19)	H9A—C9—H9C	110 (2)
C3—C2—H2B	111.2 (19)	H9B—C9—H9C	106 (2)
C1—C2—H2B	108.9 (17)	C8—C10—H10A	108.3 (18)
H2A—C2—H2B	110 (3)	C8—C10—H10B	111.7 (19)
N1—C3—C5	109.2 (2)	H10A—C10—H10B	107 (2)
N1—C3—C2	126.6 (2)	C8—C10—H10C	110 (2)
C5—C3—C2	124.1 (2)	H10A—C10—H10C	110 (3)
O2—C4—N2	123.3 (2)	H10B—C10—H10C	110 (3)
O2—C4—C5	130.5 (2)	C8—C11—H11A	107.7 (18)
N2—C4—C5	106.2 (2)	C8—C11—H11B	112.6 (17)
C3—C5—C4	107.13 (19)	H11A—C11—H11B	107 (2)
C3—C5—C6	124.1 (2)	C8—C11—H11C	114 (2)
C4—C5—C6	128.7 (2)	H11A—C11—H11C	105 (3)
N3—C6—C5	108.74 (19)	H11B—C11—H11C	110 (3)
			
C3—N1—N2—C4	1.0 (3)	O2—C4—C5—C6	2.0 (4)
C7—N3—C1—C2	125.7 (2)	N2—C4—C5—C6	−177.9 (2)
C6—N3—C1—C2	−63.6 (3)	C7—N3—C6—C5	−148.5 (2)
N3—C1—C2—C3	45.8 (3)	C1—N3—C6—C5	41.2 (3)
N2—N1—C3—C5	−0.9 (3)	C3—C5—C6—N3	−7.8 (3)
N2—N1—C3—C2	−178.8 (2)	C4—C5—C6—N3	170.1 (2)
C1—C2—C3—N1	162.0 (2)	C8—O1—C7—O3	−10.1 (4)
C1—C2—C3—C5	−15.7 (3)	C8—O1—C7—N3	170.38 (19)
N1—N2—C4—O2	179.3 (2)	C1—N3—C7—O3	−8.0 (4)
N1—N2—C4—C5	−0.8 (3)	C6—N3—C7—O3	−178.1 (2)
N1—C3—C5—C4	0.4 (3)	C1—N3—C7—O1	171.5 (2)
C2—C3—C5—C4	178.4 (2)	C6—N3—C7—O1	1.4 (3)
N1—C3—C5—C6	178.6 (2)	C7—O1—C8—C10	−179.8 (2)
C2—C3—C5—C6	−3.4 (4)	C7—O1—C8—C9	−62.4 (3)
O2—C4—C5—C3	−179.8 (2)	C7—O1—C8—C11	61.9 (3)
N2—C4—C5—C3	0.3 (3)		

### Hydrogen-bond geometry (Å, °)

**Table:**

*D*—H···*A*	*D*—H	H···*A*	*D*···*A*	*D*—H···*A*
N1—H1N1···O2^i^	0.99 (3)	1.77 (3)	2.748 (3)	171 (3)
N2—H1N2···O2^ii^	0.94 (3)	1.75 (4)	2.665 (3)	167 (3)
C1—H1B···O2^iii^	0.98 (3)	2.57 (3)	3.492 (3)	157 (3)
C11—H11C···O3^iv^	0.97 (3)	2.60 (3)	3.504 (3)	156 (3)

Symmetry codes: (i) *x*, −*y*+3/2, *z*+1/2; (ii) −*x*, −*y*+2, −*z*+1; (iii) *x*, *y*−1, *z*; (iv) *x*, −*y*+1/2, *z*−1/2.
